# The Impact of COVID-19 Pandemic on ESBL-Producing *Enterobacterales* Infections: A Scoping Review

**DOI:** 10.3390/antibiotics12061064

**Published:** 2023-06-16

**Authors:** Ha Thi Thao Mai, J. Luis Espinoza

**Affiliations:** 1 Department of Biochemistry, Faculty of Medicine, Can Tho University of Medicine and Pharmacy, Can Tho City 900000, Vietnam; 2 Faculty of Health Sciences, Kanazawa University, Kanazawa 920-0942, Ishikawa, Japan

**Keywords:** antibiotic-resistant bacteria, multidrug-resistant organisms, *Enterobacterales* extended-spectrum β-lactamase-producing *Enterobacterales*

## Abstract

Several studies have reported an increased frequency of colonization and/or infection with antibiotic-resistant bacteria (ARB) during the COVID-19 pandemic. Extended-spectrum beta-lactamase-producing *Enterobacterales* (ESBL-PE) are a group of bacteria with intrinsic resistance to multiple antibiotics, including penicillins, cephalosporins, and monobactams. These pathogens are easy to spread and can cause difficult-to-treat infections. Here, we summarize the available evidence on the impact of the COVID-19 pandemic on infections caused by ESBL-PE. Using specific criteria and keywords, we searched PubMed, MEDLINE, and EMBASE for articles published up to 30 March 2023 on potential changes in the epidemiology of ESBL-E since the beginning of the COVID-19 pandemic. We identified eight studies that documented the impact of COVID-19 on ESBL-E. Five studies were focused on assessing the frequency of ESBL-PE in patient-derived specimens, and three studies investigated the epidemiological aspects of ESBL-PE infections in the context of the COVID-19 pandemic. Some of the studies that were focused on patient specimens reported a decrease in ESBL-PE positivity during the pandemic, whereas the three studies that involved patient data (1829 patients in total) reported a higher incidence of ESBL-PE infections in patients hospitalized for COVID-19 compared with those with other conditions. There are limited data on the real impact of the COVID-19 pandemic on the epidemiology of ESBL-PE infections; however, patient-derived data suggest that the pandemic has exacerbated the spread of these pathogens.

## 1. Introduction

Resistance to antibiotics constitutes one of the most urgent global health problems, causing an estimate of 700,000 to several million deaths worldwide. According to the World Health Organization (WHO), by 2050, an estimated 350 million deaths could be attributed to antibiotic resistance. In clinical practice, infections caused by these pathogens are difficult to treat and are often associated with increased medical costs, prolonged hospital stays, and higher mortality rates. The acquisition of antimicrobial resistance is a naturally occurring process; however, the misuse and overuse of antibiotics in humans and animals, and deficient measures for the prevention or control of infections, contribute to promoting or accelerating this process [1,2].

Antibiotic-resistant bacteria (ARB) can acquire resistance to one or various antibiotics in different antimicrobial classes, and thus, depending on the number of antimicrobial agents and the number of antimicrobial classes to which bacteria are resistant, they can be classified into multidrug-resistant bacteria (MDRB), extensively drug-resistant bacteria (XDRB), or pandrug-resistant bacteria. Pathogens categorized as MDRB are resistant to at least one agent in three or more antimicrobial categories [1,3] and typically cause nosocomial infections, particularly among patients with serious conditions; however, in recent years, MDRB infections have been increasingly detected in the community setting [4].

Extended-spectrum β-lactamase-producing *Enterobacterales* (ESBL-PE) are a group of bacteria that have developed resistance to multiple antibiotics, including penicillins, cephalosporins, and monobactams, due to the production of beta-lactamase enzymes, which hydrolyze the β-lactam ring open, deactivating the molecule’s antibacterial properties, thus resulting in adverse clinical outcomes since beta-lactam antibiotics are typically used to target a broad spectrum of Gram-positive and Gram-negative bacteria. ESBL-PE tend to be resistant to other antibiotic groups, such as fluoroquinolones, trimethoprim, and tetracyclines [5,6].

ESBL-PE, especially *Escherichia coli* (*E. coli*)-producing ESBL (Eco-ESBL) and *Klebsiella pneumoniae* (*K. pneumoniae*)-producing ESBL (Kp-ESBL), are especially concerning pathogens given their potential to rapidly spread in healthcare and community settings. The transmission of ESBL-PE can occur via direct contact with infected patients or contaminated surfaces, or through the ingestion of contaminated food or water. Risk factors for colonization or infection with ESBL-PE include recent antibiotic use, hospitalization, residence in a long-term care facility, and exposure to healthcare environments [7]. Common clinical infections caused by ESBL strains include pneumonia, bacteremia, urinary tract infection (UTI), cholecystitis, cholangitis, and traveler’s diarrhea, as well as neonatal meningitis and infected wounds [8]. Some of these infections can be associated with high mortality, particularly among patients who develop severe sepsis or septic shock. Carbapenems are, in general, regarded as the preferred agent for the treatment of infections due to ESBL-producing organisms. Carbapenems are currently considered the first-line therapy, although the spread of resistant strains is a growing concern [9,10].

A critical aspect of clinical infections caused by MDRB, including ESBL-PE, is the presence of colonization, where the pathogens can be isolated from individuals in the absence of clinical symptoms [11,12]. Colonization, which typically occurs in the intestinal tract of the affected individual, is imperative and can be documented before the development of a clinical infection. Importantly, individuals colonized with ESBL-PE can spread those pathogens to other patients [13,14], which supports the crucial importance of targeting ESBL-PE colonization to prevent the dissemination of infections caused by those pathogens.

The COVID-19 pandemic caused by the severe acute respiratory syndrome coronavirus 2 (SARS-CoV-2) has disrupted healthcare and emergency services and led to a global shortage of many goods and services. Several studies have reported that infections caused by antimicrobial-resistant pathogens, including those caused by ESBL-PE, have been increasingly detected during the COVID-19 pandemic, likely in association with the high rate of empirical antibiotic utilization in COVID-19 patients, increased use of antiseptics and biocides, and the disruption of healthcare services [15,16,17,18]. Therefore, considering the clinical and epidemiological relevance of the emergence and spread of ESBL-PE in healthcare and community settings, it is important to identify potential changes in the frequency of infections caused by these pathogens as a result of the COVID-19 pandemic. Here, we performed a scoping review to summarize the available evidence on the impact of the COVID-19 pandemic on ESBL-PE infections. We utilized specific search criteria on PubMed, MEDLINE, and EMBASE, to identify studies that investigated potential changes in the epidemiology of ESBL-PE during the COVID-19 pandemic.

## 2. The Epidemiology of ESBL-PE before the COVID-19 Pandemic

The first ESBL-PE strains were reported in Germany in the 1980s, and the initial isolates were identified in clinical samples from patients who had been treated with third-generation cephalosporin antibiotics [19]. Notably, the spread of ESBL-PE was initially limited to healthcare settings; however, by the 1990s, community-acquired infections caused by these bacteria were being reported in various parts of the world, and temoneira (TEM) and sulfhydryl variable (SHV) types were the most predominant ESBL enzymes detected globally [20,21]. Reports from the early 2000s showed that the prevalence of ESBL-PE had significantly increased worldwide, and by that time, cefotaximase (CTX-M) had become the predominant ESBL enzyme worldwide, with particularly high rates of detection in Europe, Latin America, and the Asia–Pacific region [22,23]. Currently, CTX-M-type enzymes (especially CTX-M-15 variant) remain the most commonly isolated ESBL type, followed by TEM and SHV [5].

The epidemiology of infections caused by ESBL-PE is strongly influenced by geographic factors, varying across countries in the same region and even within one country. For example, a higher frequency of Eco-ESBL has been reported in Southeast Asia, Africa, and Central America compared with Europa [5,24]. Similarly, the rates of ESBL-PE are higher in northern regions than in other parts of Taiwan [25], and a study from the USA (overall prevalence of 11%) showed that while ESBL-PE prevalence was 5% in Michigan, in Washington, DC, it was 26% [26]. In terms of patient populations, infections caused by ESBL-PE are most commonly seen in hospitalized patients, particularly those with underlying medical conditions, those with immunocompromised status, and those who have been exposed to invasive medical procedures or devices, such as those in intensive care units. However, community-acquired infections are also a concern, particularly in regions with high rates of antibiotic use, where the selective pressure for resistant strains to emerge is higher [5,24,27].

A meta-analysis published in 2016 that included 66 studies and 28,909 healthy individuals revealed a pooled prevalence of ESBL class A colonization of 14%, with an increasing trend of 5.38% annually. A higher pooled prevalence was observed in Asia and Africa (ranging from 15% to 46%), while the pooled prevalence in Europe and the Americas ranged from 2% to 6%. Antibiotic use for the prior 4 or 12 months was associated with a high colonization risk, and international travel was also strongly correlated with ESBL-PE colonization [28].

A more recent systematic review of 139 publications (published up to October 2020) on the epidemiology of Eco-ESBL and Kp-ESBL in the Greater Mekong Subregion (Cambodia, Laos, Myanmar, Thailand, Vietnam, and some regions of China) showed an increasing trend in ESBL-PE detection in clinical samples and carriage of *E. coli* isolates, suggesting that ESBL-PE is widespread and dominant in both nosocomial and community settings. A meta-analysis of 12 selected studies revealed that recent exposure to antibiotics such as third-generation cephalosporin and fluoroquinolone was the most studied variable and showed a significant positive association with ESBL-E isolation, followed by chronic kidney disease and other co morbidities [27]. Similarly, a high prevalence of Eco-ESBL (42.5%) (4706/11,065) and Kp-ESBL (30.2% (1697/5617) isolates was reported in a large study conducted in northern Thailand between 2016 and 2020. The majority of ESBL-PE were discovered in airway fluids and urine, and they were highly resistant to fluoroquinolones, ampicillin, cefazolin, cefotaxime, and trimethoprim/sulfamethoxazole [29].

In recent years, international travel has been identified as a major risk factor for the acquisition of ARB, including ESBL-PE. It has been estimated that 21 to 51% of healthy travelers acquire MDR Enterobacteriaceae when traveling abroad, depending on the visited region. Thus, traveling to South Asia is associated with up to 85% acquired colonization, and colonization after traveling to Africa or the Middle East ranges from 13 to 44% [30]. A prospective study conducted in the Netherlands showed that 34% of individuals who were ESBL-PE negative before traveling to several countries worldwide had acquired ESBL-PE. Among them, those visiting southern Asia were more likely to acquire ESBL-PE pathogens, and the usage of antibiotics during travel, the presence of persistent diarrhea, and pre-existing chronic bowel disease were the strongest predictors for bacterial acquisition. Notably, about 10% of travelers remained colonized with ESBL-PE at 12 months, and had a 12% of probability of transmitting ESBL-E to a household member [31], which substantiates the importance of international travel in the acquisition and spread of ESBL-PE pathogens.

On the other hand, various reports indicate that alterations in the gut microbiome composition (dysbiosis) may promote the colonization and growth of ARB and MDRB, which appears to also be the case for ESBL-PE colonization. For example, one study showed that the presence of a higher relative abundance of *Prevotella copri* before and after traveling correlated with the occurrence of diarrhea and the acquisition of MDR *Enterobacteriaceae* after traveling to tropical regions [32]. Similarly, traveling associated with a higher frequency of antimicrobial resistance genes among bacterial isolates and a greater proportion of *Escherichia* species present in the gut microbiota [33]. On the other hand, individuals who had low *Actinobacteria* richness and low abundance of short-chain fatty acid-producing bacteria in the gut before travel had an increased risk of acquiring ESBL-PE, and the risk was particularly higher among those who ate seafood during travel [34].

**Figure 1:**
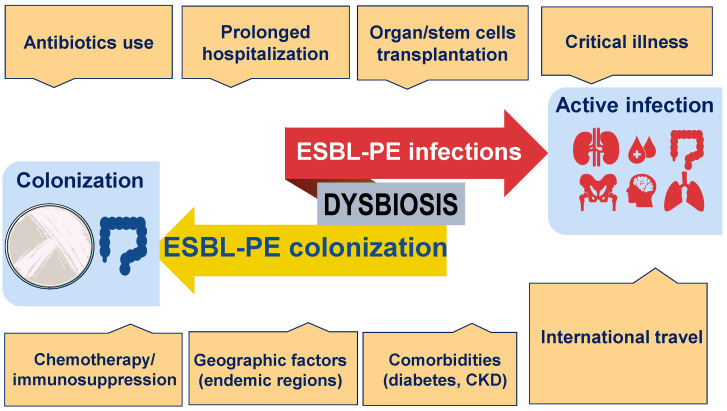
Factors that contribute to the colonization or infection with ESBL-PE.

Taken together, the available evidence indicates that the frequency and spread of ESBL-PE are determined by numerous factors, both locally and globally, and among them, the use of antibiotics, international travel, and microbiome composition appear to be major risk factors (Figure 1).

Colonization refers to the presence of ESBL-PE pathogens (often from the gastrointestinal tract) in the absence of clinical symptoms. In most patients, an active infection caused by ESBL-PE is preceded by colonization. The prevalence of both colonization and active infection by ESBL-PE is influenced by geographic factors. Other factors include extensive exposure to antibiotics; comorbidities such as diabetes, cancer, and chronic kidney disease (CKD); immunosuppression; and prolonged hospitalization. In addition, international travel and intestinal dysbiosis, which promotes the overgrowth of unwanted bacteria, have emerged as critical factors contributing to the increased risks of ESBL-PE acquisition.

## 3. The Impact of the COVID-19 Pandemic on Multidrug-Resistant Infections

During the pandemic, there was an increase in the isolation of multidrug-resistant organisms (MDROs), including Kp-ESBL, carbapenem-resistant *Enterobacterales*, *Acinetobacter baumanii* (*A. baumanii*), methicillin-resistant *Staphylococcus aureus* (MRSA), pan-echinocandin-resistant *Candida glabrata*, and multi-triazole-resistant *Aspergillus fumigatus*, and although various factors may be implicated, the increased antimicrobial use during the pandemic appears to play a major role in the spread of these pathogens [16,35,36,37]. Sulayyim et al. systematically reviewed a total of 23 articles that reported an increase in the incidence of MDRO during the COVID-19 pandemic. The authors observed that self-antibiotic treatment, antimicrobial therapy treatment, and prescriptions administered by general practitioners were risk factors associated with a higher risk of resistance during COVID-19. The study found that *A. baumannii* was the most frequently documented resistant Gram-negative bacteria, followed by *K. pneumonia*, *E. coli*, and *Pseudomona aeruginosa* (*P. aeruginosa*). Importantly, *K. pneumonia* isolates showed considerable resistance to colistin. Among Gram-positive pathogens, the most common organisms reported were *S. aureus* and *enterococcus faecium* (*E. faecium*). Importantly, a high frequency of resistance to ampicillin, azithromycin, and ciprofloxacin was documented among *E. faecium* isolates [38].

## 4. Did the COVID-19 Pandemic Affect the Epidemiology of ESBL-PE-Associated Infections?

We investigated potential changes in the rate of infections caused by ESBL-PE that could be associated with the COVID-19 pandemic. Using defined search criteria, we identified a total of eight studies, which are discussed below.

An observational study conducted between April 2020 and December 2021 in a hospital in French Guiana to assess the impact of antibiotic prescriptions on the acquisition of ESBL-PE in ICUs during the COVID-19 pandemic reported that ESBL-PE carriage ranged from 10% to 22% among ICU patients hospitalized during that period, and *K. pneumoniae* (58.3% to 84.6%) was the ESBL-PE most frequently isolated. Surprisingly, the investigators observed that exposure to cefotaxime was the only factor independently associated with ESBL-PE acquisition among ICU patients with severe COVID-19 [39].

Lemenand et al. investigated the impact of the COVID-19 pandemic on the epidemiology of Eco-ESBL in France. After analyzing clinical samples from primary care patients and nursing home residents collected between January 2019 and December 2020, they found an intriguing reduction in the percentage of Eco-ESBL isolates. Thus, while 3.1% of *E. coli* isolates in the primary care setting before March 2020 were ESBL-PE, 2.9% of *E. coli* isolates collected as of May 2020 were ESBL-PE. Similarly, in nursing homes, the Eco-ESBL rate was 9.3% before March 2020 and decreased to 8.3% after May 2020. Of note, the reduction rate accelerated from −0.04%/month to −0.22%/month from May 2020, with a sustainable reduction in Eco-ESBL rates (−0.07%/month, *p* < 0.001) after the lockdown [40]. Although several limitations were associated with this study, including the lack of contextual information to determine the relative contributions of factors such as decreased healthcare utilization, reduction in antibiotic dispensing, and the fact that the study only explored the proportion of Eco-ESBL rates and no other micro-organisms, it is interesting to observe such an apparent favorable impact of the national COVID-19 pandemic response on the Eco-ESBL epidemiology in primary care and nursing homes in France.

A case-control study from Italy reported a significant reduction in the incidence of total MDR bacterial infections during the pandemic compared to pre-pandemic years. However, among patients hospitalized in COVID-19 wards, the incidence of MDR bacterial infections was significantly higher compared with those admitted in other medical departments (29% and 19%, respectively), and Kp-ESBL was the pathogen presenting the highest increase. Notably, although the incidence rate of Kp-ESBL infections in non-COVID-19 wards decreased significantly during the pandemic (9.4 cases per 100 discharges before the pandemic vs. 4.8 cases per 100 discharges during the pandemic), it rather increased among patients hospitalized in COVID-19 departments (10.6 cases per 100 discharges) [41]. This study suggests that using pandemic-related infection-preventive measures could help tackle the spread of MDRB; however, the relative high frequency of ESBL-PE among COVID-19 patients may be associated with the underlaying disease and the extensive usage of antibiotics in these patients.

Wardoyo et al. in Indonesia studied the antibiotic susceptibility of *E. coli* from clinical specimens isolated before the pandemic (September 2019 to March 2020) and during the pandemic (March 2020 to September 2020) and reported that while the prevalence of Eco-ESBL specimens was 50% before the pandemic, it dropped to 20.9% during the pandemic. In addition, the authors observed that while sensitivity to ofloxacin, aztreonam, and fosfomycin appears to have increased during the pandemic, susceptibility to other antibiotics such as piperacillin, amoxicillin, cefadroxil, and ampicillin was significantly reduced [42].

To assess antimicrobial resistance among *E. coli* and *P. aeruginosa* isolates before and after COVID-19, Mena et al. in the Dominican Republic retrospectively examined susceptibility data from *E. coli* strains isolated from urine (27,718 cultures) and *P. aeruginosa* isolates from bodily fluids (2111 cultures) from 2018 to 2021. The study found that the Eco-ESBL in urine samples slightly decreased during the pandemic compared with the pre-pandemic period (24.75% and 25.63%, respectively), and resistance rates to carbapenems in urine samples increased from 0.11% (pre-pandemic) to 0.20%. The average rates of carbapenem resistance among *P. aeruginosa* isolates in bodily fluid also increased (2.33% vs. 3.84%) during the pandemic period [43].

Another study from Indonesia characterized the pathogens, antibiotic susceptibility patterns, and risk factors for mortality in hospitalized COVID-19 patients. Overall, the prevalence of secondary pulmonary bacterial infections in COVID-19 patients was 8.2%, and Gram-negative bacteria (64.8%), including *A. baumanii* (31.9%), *K. pneumoniae* (19.8%), and *P. aeruginosa* (8.8%), were the most commonly isolated pathogens. Importantly, 84% of *A. baumanii* isolates were resistant to carbapenem (CR-Ab), and 61.1% of *K. pneumoniae* isolates were ESBL-PE. The investigators also observed that secondary bacterial infections were associated with a higher risk of mortality, and a large number of these infections were caused by MDRB. This study substantiates the impact of MDRB, including ESBL-PE, in terms of morbidity and mortality among COVID-19 patients. However, one limitation associated with this study was the lack of data regarding the frequency of the isolated pathogens during the pre-pandemic period [44].

The incidence of secondary bacterial infections and antimicrobial resistance in COVID-19 patients was evaluated in a study from Turkey. In total, 3532 patients (4859 positive culture results) were analyzed. Among 1447 COVID-19 patients, 52 patients (3.59%) had 78 secondary bacterial infections. Interestingly, there was a significant decrease in ESBL-PE (8.94%) among COVID-19 patients compared to samples from the pre-pandemic period (20.76%). However, the rate of ESBL-PE infections among non-COVID patients (20.74%) hospitalized during the pandemic was similar to samples from the pre-pandemic. Notably, in this study, the incidence of respiratory infections caused by *A. baumanii* was significantly higher among COVID-19 patients (9.76%) compared with those hospitalized during the pre-pandemic (3.49%) and pandemic era control groups (3.11%) [45]. These findings are highly relevant considering that respiratory infections caused by this pathogen have been associated with high mortality rates [46,47].

A recent study from Canada investigated potential changes caused by the COVID-19 pandemic in the frequency of ESBL detection in urine cultures collected from community settings and long-term care (LTC) facilities. After analyzing data from 8.6 million urine cultures performed from 2016 to 2021, the investigators found that among 2.3 million positive samples, the most common isolated pathogen was *E. coli* (48.9%), followed by *K. pneumoniae* (7.2%). Importantly, 5.8% of *E. coli* and 3.3% of *K. pneumoniae* isolates were categorized as ESBLs. Overall, the study found a higher isolation rate of ESBL during the pandemic than in the pre-pandemic period; however, a regression analysis revealed that the monthly rates of ESBL detection tended to decrease during the pandemic compared with the pre-pandemic period for Eco-ESBL in both the community and LTC facilities and for Kp-ESBL in the community setting. Notably, however, in LTC facilities, the detection rate of Kp-ESBL increased during the COVID-19 period [48].

After summarizing the available literature on the impact of the COVID-19 pandemic on colonization or infections caused by ESBL-PE (Table 1), we found that two studies were conducted in Indonesia, and one each from Canada, France, Italy, Turkey, Dominican Republic, and French Guiana. All eight studies published so far were retrospective studies. Three studies (1829 patients), in addition to the frequency of ESBL-PE, also provided clinical and epidemiological aspects of the studied patients [39,41,44], while the remaining five studies were solely focused on the presence or lack of ESBL-PE in patient-derived specimens (mainly urine samples) and provided no clinical data from the affected patients [40,42,43,45,48]. The largest study that involved patients was conducted in Italy (1617 patients) and reported a higher incidence of ESBL-PE among patients hospitalized for severe COVID-19 (mainly caused by Kp-ESBL) compared with those admitted for other conditions, suggesting that COVID-19 may predispose patients to colonization and thereby infection with ESBL-PE, likely due to the higher frequency of these patients undergoing invasive procedures, such as invasive ventilation and long-term exposure to catheters [41].

In the last two decades, numerous studies have shown that alterations in the gut microbiota composition are implicated in the colonization with MDRB, including ESBL. Thus, restoring microbiota composition can be helpful to eradicate ESBL colonization. In this regard, various studies have tested the clinical utility of restoring gut microbiota composition to eradicate colonization with ESBL-PE by utilizing dietary interventions, antibiotic stewardship, or administration of probiotics [49,50,51,52], and more recently with the use of fecal microbiota transplantation (FMT), based on the successful experience using this approach to combat *Clostridioides diffile* infections [53]. Indeed, several studies have reported the successful eradication of ESBL-PE in various clinical settings, including in patients with immunosuppressive conditions, such as in those under chemotherapy for cancer or in recipients of hematopoietic stem cells transplantation [11,54].

In a recent study conducted in Hong Kong, the authors observed that as a result of the stricter measures associated with the COVID-19 pandemic and due to the addition of stringent screening for ESBL-PE amongst potential FMT donors, there was a dramatic decrease in successful FMT donor recruitment rates, dropping from 6.7% before the pandemic to 0.8% during the pandemic. Importantly, among the 119 prospective stool donors, a large proportion (86%) failed stool testing due to positivity for ESBL-PE, and only one potential donor out of 119 (0.8%) was effectively enrolled as a recurring contributor [55].

There are some limitations associated with this study. Although we conducted robust systematic searches in three relevant databases, studies not published in English were excluded, which may have resulted in relevant studies being missed. In addition, during the search process, we did not consider unpublished and grey research (conference abstracts or any informally published material) to identify unpublished data. Additionally, considering that COVID-19 is an evolving disease and new studies are emerging rapidly, we cannot exclude the possibility that different trends in ESBL-PE infections associated with the pandemic could be depicted in new publications.

## 5. Concluding Remarks

**Figure 2:**
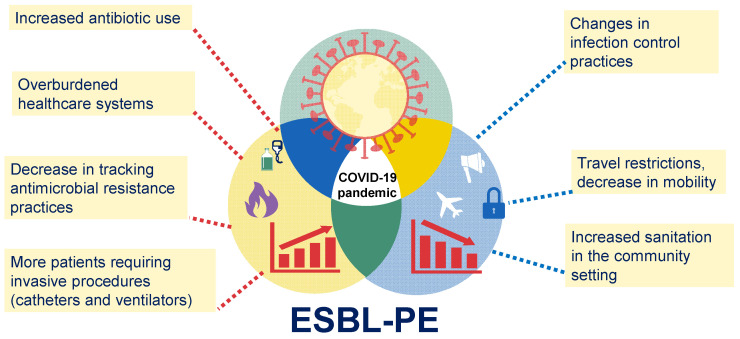
The COVID-19 pandemic and its potential effects on ESBL-PE infections.

There are limited data on the real impact of the COVID-19 pandemic on the epidemiology of ESBL-PE infections, with some conflicting results, where some studies (especially those focused on specimens and not patients) report a decrease in the frequency of ESBL-PE in specimens collected during the COVID-19 pandemic, while other studies (especially those focused on patient’s data) report an increase in the incidence of ESBL-PE infections during the COVID-19 pandemic. The emergence and propagation of ARB constitute one of the leading public health threats of the current century, and accumulating evidence indicates that this problem has been aggravated during the COVID-19 pandemic. Due to the multiple challenges faced by healthcare systems during the pandemic, various factors may have contributed to the spread of antibiotic-resistant pathogens, including ESBL-PE. For example, the increased use of antibiotics during the pandemic may have contributed to the emergence and spread of antibiotic-resistant bacteria, including ESBL-PE. Additionally, the pandemic has placed a significant strain on healthcare systems worldwide, leading to overcrowding, shortages of essential supplies, and an increased risk of infection transmission, thereby contributing to the spread of ESBL-PE in healthcare settings. In addition, changes in infection control practices to manage the COVID-19 pandemic may have had a dual effect on the emergence of ESBL-PE. On the one hand, many healthcare facilities have implemented changes in infection control practices, such as the use of personal protective equipment and increased cleaning and disinfection measures. While these measures are important for preventing the spread of COVID-19, they may also impact the spread of other infectious agents, including antibiotic-resistant bacteria (Figure 2).

Various factors associated with the COVID-19 pandemic may have contributed to an increase or decrease in the prevalence of ESBL-PE infections. For example, the increased use of antibiotics during the pandemic, the large number of patients in critical condition, and the overwhelmed healthcare systems may have increased the risk of the emergence and spread of ESBL-PE. Conversely, changes in infection control practices implemented in many healthcare settings and even in the community and international travel restrictions may be associated with a decreased risk of propagation of ESBL-PE.

In conclusion, while the impact of the COVID-19 pandemic on the epidemiology of ESBL-PE infections is not yet fully understood, it underscores the importance of continued efforts to address the problem of antibiotic resistance, including the development of new antibiotics and alternative therapies, improved infection control measures, and the importance of antibiotic stewardship programs.

## 6. Materials and Methods

This scoping review was conducted according to the Preferred Reporting Items for Systematic Reviews and Meta-Analyses Extension for Scoping Reviews (PRISMA-ScR) Statement [56]. The scoping methodology was preferable for this study because of the high degree of heterogeneity of the included research. A literature search was performed using PubMed, MEDLINE, and EMBASE through to 31 March 2023. References to retrieved articles were manually searched to ensure the identification of studies not found in the initial literature search. The selection was limited to publications written in English.

### 6.1. Eligibility Criteria

The inclusion criteria for articles were as follows: studies reporting data from patients with COVID-19 or hospitalized in COVID-19 wards, or from patient-derived specimens obtained during the COVID-19 pandemic. We included in the analysis only peer-reviewed original articles that were published in the English language. An initial search of publications was performed between 20 December 2022 and 12 January 2023. Next, taking into account the rapid increase in the scientific literature on the COVID-19 pandemic between 20 March and 31 March 2023, we performed a second and updated search of the literature in order to identify new and potentially relevant evidence. We used standardized search terms, MeSH (medical subject headings) in MEDLINE and PubMed, and Emtree in Embase, organized in a hierarchal structure. The search terms were used in combination, as detailed in Table 2.

### 6.2. Study Selection and Extraction

The article search and study selection were completed by two independent reviewers (JLE and HTTM). The article search included a pilot test of screening for the first 50 search results to standardize the search criteria. Each abstract underwent three rounds of evaluation by a separate reviewer. Reviewers independently screened titles and abstracts and full texts to identify all potentially relevant studies. Any discrepancies during article screening were resolved through consensus between the two reviewers (JLE and HTTM). Data extraction was performed independently by the two reviewers (JLE and HTTM).

### 6.3. Categorization and Analysis

**Figure 3:**
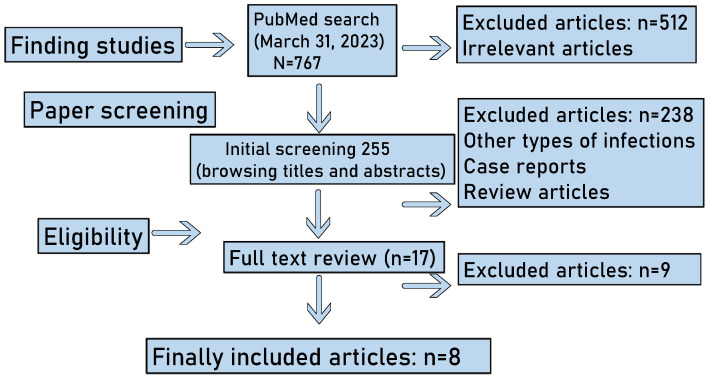
Outline of the literature search. Our search yielded a total of 767 articles, and 512 were rapidly excluded because they were considered irrelevant. The remaining 255 articles were further screened for titles and abstracts, and thereafter, 238 of them were excluded because some of them were case reports, some studies were review articles, and some reported other types of infections. Finally, 17 articles were subjected to a full text review, and 8 of them were found to fulfill the inclusion criteria and were therefore included in this scoping review.

We categorized the studies into those that included patients’ clinical data and those that only examined patient-derived specimens. The literature search strategy and outcomes are summarized in Figure 3.

## Figures and Tables

**Table 1:** Demographics and epidemiological characteristics of the reported studies.

Reference Author/Country/ Journal	No of Subjects/Specimens	Type of Study	Bacterial Strains	Main Findings
G. Ngoula, 2023, French Guiana. Antibiotics [39]	311 patients	Observational study	*K. pneumonieae* *E. Coli* *E. cloacae* *K. aerogenes*	22.8% of ICU patients had ESBL-PE. Risk of ESBL-PE carriage among patients with severe COVID-19 was higher when they were exposed to cefotaxime.
O. Lemenand, 2021, France. J Infection [40]	793,954 *E. coli* isolates from 1022 clinical laboratories	Retrospective multicenter study	*E.coli*	In general practice, Eco-ESBL decreased lightly during the pandemic (3.1% before vs. 2.9% during the pandemic). In nursing homes, the Eco-ESBL rate decreased from 9.3% to 8.3%.
E. Bentivegna, 2021, Italy, Int J Environ Res Public Health. [41].	1617 patients	Case-control study	*S. aureus*, *K. pneumoniae*, *C. difficile*, and *A. baumannii.*	Significant higher incidence of MDRB infections in COVID-19 departments than in other medical departments (29% vs. 19%); Kp-ESBL was the pathogen with the highest increase.
E. Wardoyo, 2021, Indonesia. Iran J Microbiol [42].	210 *E. coli* isolates	Retrospective single center study	*E. coli*	Among *E. coli* specimens isolated before the pandemic, 50% were Eco-ESBL and 21% of those collected during the pandemic were Eco-ESBL.
A. Mena, 2022, the Dominican Republic. Antimicrob Steward Health Epidemiol [43].	27,718 urine cultures and 2111 body fluid cultures	Retrospective study	*E. coli* *P. aeruginosa*	The frequency of Eco-ESBL was 25.63% before and 24.75% after the COVID-19 pandemic.
P. Santoso, 2022, Indonesia. Int J Gen Med [44].	182 patients	Observational study in two hospitals	*A. baumanii*, *P. aeruginosa* *K. pneumoniae*	45.9% of COVID-19 isolates were MDRB, including CR- *A. baumannii* (84%) and Kp-ESBL (61%).
M. Karataş, 2021, Turkey. Ann Clin Microbiol Antimicrob [45].	Total N = 4859 isolates. Pre-pandemic: 3034 isolates. Pandemic non-COVID: 1702 isolates. COVID-19 patients: 123 isolates.	Retrospective single-center study	*E. coli* *K. pneumonieae A. baumannii* *S. aureus*	ESBL-PE infections were less common in isolates from COVID-19 patients (8.94%) compared to pre-pandemic samples (20.7%) and samples from non-COVID-19 patients collected during the pandemic (20.7%). Among COVID-19 patients, *E. coli* was rarely detected, but *A. baumannii* was more commonly found than in controls.
M.R. Hasan, 2023, Canada, Microbiol Spectrum [48].	8,652,381 urine cultures	Retrospective, observational study	*Eco-ESBL* *Kp-ESBL*	The rate of ESBL isolation was higher during the pandemic than before it. However, decreasing trends in both Eco-ESBL and Kp-ESBL in the community setting were observed during the pandemic.

**Abbreviations**: *A. baumanii*: *Acinetobacter baumanii*; CR: carbapenem resistant; ESBL: extended-spectrum beta-lactamase; ESBL-PE: ESBL-producing *Enterobacterales*; *E. cloacae*: *Enterobacter cloacae*; *K. pneumoniae*: *Klebsiella pneumoniae*; *K. aerogenes*: *Klebsiella aerogenes*; Kp-ESBL: extended-spectrum beta-lactamase *Klebsiella pneumoniae*; *P. aeruginosa*: *Pseudomonas aeruginosa*; *Eco*: *Escherichia coli*; Eco-ESBL: extended-spectrum beta-lactamase-producing *Escherichia coli*; *C. Difficile*: *Clostridioides difficile*; MDRB: multidrug-resistant bacteria; *S. aureus*: *Staphylococcus aureus*.

**Table 2:** The search terms for the literature review.

Topic	Search Terms
Context	COVID-19 COVID-19 pandemic SARS-CoV2 Coronavirus pandemic
Bacteria	*Enterobacterales* AND (ESBL OR ESBL-positive OR ESBL-producing OR *Enterobacteriaceae* OR extended-spectrum beta-lactamase OR extended spectrum beta lactamase OR extended spectrum beta lactamases)
Outcomes	‘COVID-19 ESBL’ OR ‘COVID-19 extended-spectrum beta-lactamase’ OR ‘pandemic associated esbl’ OR ‘pandemic associated ESBL’ OR ‘COVID-19 *Enterobacterales*’ OR ‘COVID-19 enterobacterales’ OR ‘COVID-19 enterobacteriaceae’

## Data Availability

Not applicable.
